# National Health Insurance Scheme: An Assessment of Service Quality and Clients' Dissatisfaction

**DOI:** 10.4314/ejhs.v30i5.20

**Published:** 2020-09

**Authors:** Ajeigbe Abiodun Kofoworola, Ayinbuomwan Ekiye, Adesina Olufunlola Motunrayo, Adedeji Tewogbade Adeoye, Makinde Ronke Adunni

**Affiliations:** 1 Department of Chemical Pathology, Obafemi Awolowo University (OAU), Ile-Ife; 2 Department of Chemical Pathology, University of Benin (UNIBEN), Benin-City; 3 Department of Oral and Maxillofacial Surgery/Oral Pathology, OAU, Ile-Ife

**Keywords:** National Health Insurance Scheme, Dissatisfaction, Service delivery

## Abstract

**Background:**

Health Insurance Scheme (NHIS), a medical package to start with a fraction of Nigerians at its inception, with the expectation of optimal services to all in the long The Nigerian government established National run. However, there are complaints and dissatisfaction of enrolees of the scheme. This study determined factors responsible for enrolees' dissatisfaction of services in a general hospital within the federal capital territory, Abuja.

**Method:**

Three hundred enrolees of National Health Insurance Scheme at the Kubwa general hospital were enrolled. Semi structured questionnaires were used to obtain information on socio-demography, education, enrolee status, perception of the scheme and factors responsible for enrolees' dissatisfaction. SPSS version 22 was used to analyse Data using percentage.

**Results:**

Majority (66.3%) of the respondents were between 35 and 54 years while 28.5% were below 35years and 11.8% (32) were above 54years with the male to female ratio was 1.03:1. Sixty percent (179) of the respondents had a minimum of tertiary education with just 1.8% having primary education. Most (69.9% and 79.6%) respondents were principal enrolees and public servants respectively. Seventy percent of the respondents have been enrolled in NHIS for more than 3years and had a good perspective of the scheme. However, 30% of the respondents were not satisfied with NHIS services with 8.6% and 15.4% describing the services as substandard and below expectations respectively. Half (50.7%) of the respondents would consider an alternative to NHIS suggesting their dissatisfaction. The major factors responsible for dissatisfaction were billing system, long waiting hours and staff attitude accounting for 46.9%, 59.4% and 7.8% respectively.

**Conclusion:**

This study revealed that the level of clients' dissatisfaction with NHIS services is high despite their acceptance of the scheme with the major areas of concern being the billing system, waiting time and staff attitude. Therefore, it is necessary for the providers to look more into these areas as targets for service delivery improvement.

## Introduction

Provision of free health for all is a major challenge in Nigeria. Although government officials and students have long benefitted from subsidized healthcare services, the general population still pay for these services. Since the introduction of National Health Insurance Scheme (NHIS) in 2005 (1) based on contributory finance, it is believed that the pooled funding would cater for infrastructure, manpower and equipment necessary for clients' health needs (2). However, the clients' experience of NHIS services over the years is unsatisfactory as the majority of enrolees are displeased and not satisfied with services rendered. Therefore, this has resulted in accessing alternative health facilities where outright out of pocket (OOP) payment would be made. Thus, it is important to identify those areas causing clients' dissatisfaction as targets for service improvement. The need for NHIS in Nigeria was first recognized in 1962 (1,3), approved in 1997, passed into law in 1999 and officially launched on 6^th^ June 2005. The objective of NHIS is to provide comprehensive healthcare at affordable costs with limited percentage of citizens covered (government and private employees, vulnerable groups). Based on this, it is important to measure health system effectiveness as it aligns to improvements in access to quality healthcare and client satisfaction (4). Patient satisfaction is defined as patient's judgement on the quality and goodness of care. It is the best possible health outcome given the available resources consistent with patient values and preferences. Patients, being the healthcare services consumers, have expectations and needs warranting satisfaction (5). This determines the quality of care by healthcare providers which is a professional responsibility. NHIS enrollees' dissatisfaction in Nigeria is yet to be fully explored necessitating the need to evaluate causes and factors associated with enrolees' dissatisfaction of healthcare services using selected indices of satisfaction in the hospital. This is with the view of improving service delivery within the hospital settings

Previous studies evaluating the NHIS performances reported that there were complaints where providers denied enrolees their full entitlements and some providers charged additional fees (6–8). Another study described enrolees' experiences as unsatisfactory with inadequate drugs, poor registration services and referral system as well as delayed services as reasons (7) for their dissatisfaction. It has been found that waiting time and attitude of healthcare staff were predictors of users' satisfaction of NHIS (9). Similarly, in the evaluation of NHIS in 2006, the major areas of clients' dissatisfaction were poor attitude and behaviour of service providers (10). Another study in Ibadan reported that 54.7% of their respondents were dissatisfied with the process of enrolment, 50.5% with range of services, 62.1% with the co-payment plan, 55.4% with change of provider and an overall dissatisfaction of 16.4% (11). The report from Jos was that 26% of their respondents were dissatisfied, necessitating an immediate attention from providers and Health Maintenance Organizations (HMOs), with sources of dissatisfaction being poor registration services, poor referral system, delay of services and unavailable services (12). Prolonged waiting time was a major cause of dissatisfaction with observations of inefficiency and ineffectiveness in some areas of operations (7,11). A similar study in Enugu by Iloh reported that bureaucracy with prolonged waiting time was a major concern (13). Patient satisfaction being a dominant concern mentioned with strategic health services decisions (WHO) requires evaluation. Surveys on satisfaction have been widely used to address access and performance (14–16) which has helped government agencies to measure performance (17). It has been suggested that there is a need for continuous monitoring of clients' satisfaction and dissatisfaction (18) through research to impact future implementation strategies which can be imported to the healthcare system. We therefore assessed the factors responsible for NHIS enrolees' dissatisfaction in a general hospital setting at the Federal Capital Territory which is supposed to be a pace setter of quality service delivery for other centres to emulate within the country.

## Methods

This is a descriptive cross-sectional study conducted at the NHIS clinic of Kubwa General Hospital (KGH), Abuja, between November 2016 and March 2017. The hospital is one of the secondary health facilities within the Federal capital development authority having a 200 bed capacity. It is a major referral centre to neighbouring general hospitals and primary health centres. The population for this study were all enrolees of NHIS between 18 and 60 years attending KGH as the primary healthcare provider. The estimated enrolees were 5000 within six months and above. Excluded from the study were those who joined the scheme less than 6 months and did not visit clinics during the period of study. The calculated sample size was 295.6 using Leslie-Kish formula: n= Zα2pq/d2 for descriptive study using 26% proportion of enrolees satisfaction of NHIS from a previous study by Onyedibe (7). A total of 300 enrolees were sampled using simple random sampling. Data was collected from consenting enrolees using questionnaires. Data was analysed with the Statistical Package for Social Sciences (SPSS) version 22. Likert scales were used to assess the degree of agreement and disagreement with statements in the questionnaire. Chi-square was used to assess the relationship between the variables of dissatisfaction and sociodemographic parameters, and a P< 0.05 was taken as statistically significant. Results were presented using tables. A pre-test was done to assess the component of questionnaire for data collection and respondents' reactions. Ethical clearance was obtained from the Ethical and Research Committee of KGH, Abuja, and written informed consent was obtained from respondents.

## Results

Three hundred subjects were given self-administered questionnaires to fill with 93% response rate. Enrolees between 25 and 54 years of age formed the majority (90.5%) of our respondents with 4.3% below 24 years and 11.8% above 54 years reflecting that youths and middle aged constitute the bulk of the labour force in Nigeria. Males (50.9%) and females of the workforce (Table 1).

**Table 1:** Distribution of age and sex of respondents (n= 279)

Variable	Frequency (%)
**AGE (years)**	
**15–24**	12 (4.3)
**25–34**	50 (17.9)
**35–44**	102 (36.6)
**45–54**	83 (29.7)
**55–64**	32 (11.5)
**Sex**	
**Male**	142 (50.9)
**Female**	137 (49.1)

About 70% and 80% of the respondents were principal enrolees of NHIS from government establishments respectively suggesting a good health-seeking behaviour and support from government. The majority (78.1%) of the respondents were married in a monogamous 95% family setting. Less than 50% of the respondents had been enrolled for over 5 years with 58.2% enrolled for 5 years (Tables 2 and 3).

**Table 2:** Educational and NhIS enrollee status (n=279)

Variable	Frequency (%)
**Educational Level**	
Primary	5 (1.8)
Secondary	95 (34.1)
Tertiary	179 (64.2*)
**NHIS Status**	
Principal	195 (69.9)
Dependent	84 (30.1)
**Enrolment** **period(year)**	
<1	18 (6.5)
1–3	26 (9.3)
3–5	110 (39.4)
>5	125 (44.8)
**Employment Status**	
Government	222 (79.6*)
Private	13 (4.6)
Self Employed	22 (7.9)
Dependent	22 (7.9)

**Table 3:** Marital and family status of respondents (n=279)

Variable	Frequency (%)
**Marital Status**	
Single	61 (21.9)
Married	218 (78.1)
**Family setup**	
Monogamous	265 (95)
Polygamous	14 (5)
**Family size**	
<6	204 (73.1)
>6	75 (26.9)

Most (74.7%) respondents perceived services of NHIS as quality although (49.1%) were evenly distributed suggesting gender balance 22.9% were not satisfied with the services while 7.9% of the respondents were undecided. More than half (50.7%) of the respondents would prefer an alternative to NHIS while the majority wanted (88.4%) NHIS sustained and improved (Table 4).

**Table 4:** Quality and preference for NHIS services (n=279)

Variable	Frequency (%)
**Quality of Services**	
Below expectation	43 (15.4)
Substandard	24 (8.6)
Standard	177 (63.4)
Quality	35 (12.5)
**Responses to quality** **service**	
Undecided	14 (5.1)
Disagree	9 (3.2)
Agree	207 (74.7)
Strongly agree	47 (17)
Non-response	2 (0.7)
**Preference for NHIS**	
Never	0 (0)
Maybe	140 (50.7)
Definitely Yes	136 (49.3)
Non-response	3

The reasons for dissatisfaction were billing system, waiting time and staff attitude accounting for 46.9, 59.4 and 7.8% respectively. Two hundred and six of respondents spent most time waiting for consultation followed by laboratory tests (9.8%), nurses station (6.2%) and medical records (5.8%). Most (48.5%) were not pleased with the attitude of staff in records station followed by those in pharmacy (26.2%), laboratory (13.6%), nursing (6.8%) and radiology (4.9%) (Table 5).

**Table 5:** Areas of dissatisfaction (n=279)

Variable	Frequency (%)
**BILLING SYSTEM**	
Undecided	2 (0.7)
Uncomfortable	130 (46.9)
Very comfortable	145 (52.4)
Non-response	2
**Waiting Time**	
<1	109 (39.1)
1–2	10 (3.6)
2–3	22 (7.9)
3–4	138 (49.4)
**Staff Attitude**	
Undecided	2 (0.7)
Dissatisfied	21 (7.8)
Satisfied	205 (76.5)
Very Satisfied	40 (14.9)
Non-response	11

Most respondents (48.9%) would like to see an improvement on the waiting time at the pharmacy services (32.2%), laboratory (14.8%) and billing (4.2%) (Table 6). This improvement would increase the satisfaction of clients as the services would be perceived robust.

**Table 6:** Sources of dissatisfaction (n=279)

Variable	Frequency (%)
**Waiting time**	
Medical records	16 (5.8)
Nurses station	17 (6.2)
Consulting room	216 (78.3)
Laboratory	27 (9.8)
Non-response	3
**Professional sources**	
Records staff	50 (48.5)
Nursing staff	7 (6.8)
Lab staff	14 (13.6)
Pharmacy staff	27 (26.2)
Radiology staff	5 (4.9)
Medical staff	0
Non-response	176
**Prof. sources of** **satisfaction**	
All professionals	17 (7.9)
Lab staff	2 (0.9)
Records staff	26
Medical Staff	170
Non-response	64
**Areas of** **Improvement**	
Waiting Time	129 (48.9)
Pharmacy services	85 (32.2)
Lab. Services	39 (14.8)
Billing	11 (4.1)
Non-response	15

## Discussion

The age distribution of the respondents in this study clearly reflects the coverage of all age groups by NHIS in line with its objectives. It also shows that NHIS is mostly accessed by those in the working age of 25–60 years who are primary enrolees. It is also obvious that the workforce in Nigeria is between 25 and 54 years with a peak of 35–44 years. There is also a wide coverage to capture the dependents of primary enrolees. Males and females in this study had similar health seeking attitude which was good. This may be due to the impact of the educational level of the respondents in which the majority were found to have a minimum of tertiary education. This in turn might be a reflection of a high literacy level of city dwellers and because NHIS captures skilled workers in government establishments. This supports earlier findings by Kayode et al (11). In this study, it was observed that the principal enrolees were more than the dependents probably due to the fact that they were primary targets and government workers constituting more than two-third of the enrolees compared to other workers. This is similar to reports from Jos by Shaffiu (12). Although NHIS kicked off in 2015 with the formal government sector which is just less than 25% of the population with the projections of expanding the scheme to include the informal sector, it is surprising that NHIS is still struggling to provide healthcare services to a few Nigerians more than 10 years after its inception. Neglected citizens include artisans, traders, transporters, farmers and market women who are yet to benefit from NHIS. Akeem in his study among artisans in Lagos in 2011 determined the reactions of these group to NHIS and observed that these citizens felt neglected and marginalized (19). There have been reports that these groups can access NHIS services, but the package is not robust and the protocol is often ambiguous leading to frustration of individuals. This study found that NHIS was attended by married enrolees probably because of access by dependent family members. More married people had monogamous family settings with a larger family size which could help NHIS make forecast and plan for the future services. The respondents in this study have a larger percentage of enrolees of more than 3 years suggesting that they may have a better and clearer perspective of NHIS services and give better information. Kayode et al reported that clients with longer duration of enrolment were more satisfied with waiting time and staff attitude (11).

The majority of the respondents agreed to NHIS provision of quality services but some of the enrolees are still unsatisfied. Shaffiu in 2011 reported similar satisfaction (61.5%) and dissatisfaction (26%) rates supported by Iloh who reported overall satisfaction rate of 66.5% among his study participants in South-eastern Nigeria in 2016 (12–13). Similarly, Onyedibe et al reported 61.5% satisfaction with NHIS services and 26% dissatisfaction in the participants of a study in 2012 (7). However, the report from Ibadan by kayode et al revealed that fluctuating levels of satisfaction with NHIS services were dependent on components being examined (11). This study confirms earlier reports of higher rates of NHIS services satisfaction than dissatisfaction. Although, many clients appear to be satisfied with services rendered by NHIS, there is a need for necessary action to improve the clients' dissatisfaction. This is because the level of dissatisfaction of NHIS enrolees is still high and may even be higher if different separate components of NHIS services are examined independently. Thus, suggesting a need for indepth NHIS services evaluation and assessment of its different units or sections as areas for targeted improvements. It was also observed that most respondents were willing to continue patronage of NHIS in the presence of alternatives suggesting good service delivery. Also, participants found NHIS billing comfortable, and it eased the burden of OOP payment which could be difficult for salary earners. On the other hand, many participants were uncomfortable with the billing system due to frequent out-of-stock drugs in the pharmacy necessitating OOP purchase outside the service centres. This unsatisfied group felt cheated as they had fully paid their contributory fund. This suggests that these individuals could not get value for their money.

Most time was spent at the consulting room waiting to see the doctor. This is due to the fact that few doctors are now available in Nigeria. It was reported that the ratio of doctor-to-patient is 1:53,333 in Nigeria buttressing the shortage of this highly skilled professionals due to brain drain as a result of poor work conditions, lack of welfare, lack of motivation and poor remuneration. More so, NHIS clinics utilize medical officers within the domiciled hospitals who have other clinical roles. Despite the prolonged time waiting to see the doctor, it was observed that most respondents were mostly pleased with doctors' attitude followed by the attitudes of record staff and the laboratory staff the least. This supports earlier reports by Iloh and Ofili (13,20). On the other hand, half of the respondents were not pleased with the attitude of staff in records, pharmacy, laboratory, nursing and radiology units. This finding may be two way viz the staff and the client factors. This may be due to the fact that the records station is the first point of contact for most enrolees, who are usually in a haste. Often times, record staff might be unwilling to follow protocol in case file retrieval for most patients. The staff in this section also handle lots of patient information which include data on registration, retrieval/filing of new clients folder, archive of important documents, generation of approval codes for investigations and referrals as well as stamping of prescription forms. They are also saddled with responsibility of authorization of referral forms, liaisons between enrolee and HMOs among other functions. In addition, there are usually fewer personnel to man this station and handle numerous administrative roles, eventually overwhelming the staff and thus, their disposition to the enrolees may be perceived as poor. Thereby, resulting in frequent friction between enrolees and officers. The medical records, being an integral component of NHIS, require necessary attention and periodic staff performance evaluation and training to improve this area of service delivery. Many of the respondents in this study desire an improvement of NHIS services with most emphasis on the waiting time at the clinics, pharmacy and the laboratory. It is therefore important for stakeholders to look deeply into these areas and make proper adjustments.

The number of absent responses to questions requiring enrolees' comment on their satisfaction with different professional groups was low compared to the responses to other questions. This might be due to a general perception in our environment not supporting negative comments on staff especially within a public setup. It is believed that such comments could cause dismissal of such employee. Thus, clients will rather keep mute than give a contrary opinion regarding wrong attitude of public servants in discharging their duties. This may also suggest the basis of gross indiscipline and impunity in government establishments. However, if we desire an improvement in our public services, there must be healthy criticisms from users of such services. Therefore, these may be areas of great importance to future research.

This study has some limitations. One is nonrecruitment of NHIS staff members who are also enrolees or potential enrolees of the scheme. This is because of the perceived possibility of them being biased in answering the questions on satisfaction of services they rendered. Another limitation is the study being single-centred, as a multi-centred study may give a broader picture of clients' satisfaction.

In conclusion, in spite of the above stated limitations, it could be conclusively inferred from this study that evaluation of healthcare service delivery should be a continuous exercise to ensure quality service delivery or clients' satisfaction. NHIS should expand their services to capture the non-skilled workforce, and remove the bottlenecks associated with registration. It is hoped that different researchers would find NHIS service delivery as an area of further research.
